# *Parabacteroides vesiculifaciens* sp. nov., a Novel Immunomodulatory, Vesicle-Producing Gut Commensal Isolated from the Human Gut

**DOI:** 10.3390/ijms27062763

**Published:** 2026-03-18

**Authors:** Andrei V. Chaplin, Irina V. Podoprigora, Victoria A. Shcherbakova, Natalya B. Zakharzhevskaya, Peter V. Evseev, Anna A. Vasilyeva, Filipp A. Koshkin, Dmitry A. Kardonsky, Elizaveta A. Vorobyeva, Daria A. Kashatnikova, Victoriia D. Kazakova, Boris A. Efimov

**Affiliations:** 1 Lopukhin Federal Research and Clinical Center of Physical-Chemical Medicine of Federal Medical Biological Agency, Malaya Pirogovskaya St. 1a, 119435 Moscow, Russia; natazaha@gmail.com (N.B.Z.); freddy1178@yandex.ru (D.A.K.); elizavetavorobjeva@yandex.ru (E.A.V.); daria_sv11@mail.ru (D.A.K.); viktorialuzanova.1998@gmail.com (V.D.K.); efimov_ba@mail.ru (B.A.E.); 2 Pirogov Russian National Research Medical University, Ostrovityanova Str. 1, 117997 Moscow, Russia; podoprigora-iv@rudn.ru (I.V.P.); petevseev@gmail.com (P.V.E.); annavasilyeva.bioscience@gmail.com (A.A.V.); 3 Department of Microbiology Named After V.S. Kiktenko, Medical Institute, Peoples’ Friendship University of Russia Named After Patrice Lumumba (RUDN University), Miklukho-Maklaya Str. 6, 117198 Moscow, Russia; 4 Skryabin Institute of Biochemistry and Physiology of Microorganisms, Federal Research Center “Pushchino Scientific Center for Biological Research, Russian Academy of Sciences”, Prospekt Nauki 5, 142290 Pushchino, Russia; vshakola@gmail.com; 5 Independent Researcher, 119270 Moscow, Russia; koshkinfilipp@gmail.com; 6 The Laboratory of Ecological Genetics, Vavilov Institute of General Genetics, Gubkina Str. 3, Russian Academy of Sciences, 119333 Moscow, Russia

**Keywords:** *Parabacteroides*, *Tannerellaceae*, *Bacteroidota*, gut microbiota, novel species, extracellular vesicles

## Abstract

The genus *Parabacteroides* comprises widespread gastrointestinal commensals, known to produce immunomodulatory molecules and extracellular vesicles, yet its full diversity is incompletely cataloged. This study describes strain ASD2025^T^, isolated from healthy child feces, using a polyphasic taxonomic approach including phenotypic profiling, chemotaxonomy, and comparative genomics. Cells were non-motile, polymorphic rods that produced extracellular vesicles. Phylogenomic analysis placed ASD2025^T^ within the genus *Parabacteroides* within a species complex consisting of *P. acidifaciens*, *P. hominis*, “*P. massiliensis*”, *P. merdae,* and *P. johnsonii*, with average nucleotide identities to the type strains of 85.5–89.9%. The large genome (5.16 Mbp, 46.2% GC content) contained integrative conjugative elements harboring antibiotic resistance genes and hankyphage-related prophage. The strain produced succinate as the major metabolic end product, and its major fatty acids were anteiso-C_15:0_, iso-C_17:0_ 3-OH, and C_15:0_. Conditioned medium from ASD2025^T^ antagonized the interleukin-8 response caused by *E. coli* lipopolysaccharide in HT29 cells. The majority of related metagenome-assembled genomes originate from mouse microbiomes. Based on these distinct characteristics, strain ASD2025^T^ (=VKM B-3926^T^ = JCM 37967^T^) represents a novel species of the genus *Parabacteroides*, for which the name *Parabacteroides vesiculifaciens* sp. nov. is proposed.

## 1. Introduction

The genus *Parabacteroides* comprises a group of obligately anaerobic, non-spore-forming, Gram-negative bacteria within the recently established family *Tannerellaceae* of the phylum *Bacteroidota* [1]. Originally classified under the genus *Bacteroides*, it was re-evaluated and established as a distinct genus based on comparative analysis of 16S ribosomal RNA gene sequences and chemotaxonomic features [2].

These bacteria are integral members of the human gut microbiota, being detected in 95% of fecal samples from Western populations using 16S rRNA gene amplicon sequencing [3]. The average abundance of *Parabacteroides* in the gut microbiota across 12 human populations is approximately 1.27% [4,5]. The predominant species in the human intestinal metagenomic data are *P. distasonis* and *P. merdae* [6,7]. Besides humans, *Parabacteroides* species have also been isolated from calves [8], pigs [9,10] and chinchillas [11]. Metagenomic studies further suggest the presence of uncultured *Parabacteroides* clades within poultry microbiomes [12]. Genome-based taxonomy database GTDB [13] currently lists 50 species-level clades of Parabacteroides lacking species names, indicating that the diversity of the genus remains incompletely resolved.

Compared to bacteria of the *Bacteroides* genus—the predominant member of human gut *Bacteroidetes*—*Parabacteroides* species degrade a narrower spectrum of plant polysaccharides [7,14] and are capable of weak growth in a medium without the addition of carbohydrates [14], indicating distinct nutrient niche [15].

Intestinal *Parabacteroides* employ a multifaceted strategy to modulate inflammatory response. These bacteria, along with *Bacteroides* species, lack an acyltransferase LpxM responsible for adding the sixth acyl chain to lipid A within lipopolysaccharide (LPS) molecules [16,17]. The resulting hypo-acylated LPS demonstrates antagonistic activity toward TLR4-mediated immune responses, likely by competing with pro-inflammatory hexa-acylated LPS for the same binding site on MD-2 [17,18,19]. *P. distasonis* produces secondary bile acids that inhibit differentiation of Th17 cells, promote the M2 polarization of macrophages and lower intestinal barrier permeability for LPS [20,21]. The transformation of branched-chain amino acids to branched-chain short fatty acids by *Parabacteroides* species ameliorates systemic inflammation, atherosclerosis and metabolic disorders in obese mice [22,23].

*Parabacteroides* species, as well as other *Bacteroidales* members, are known to produce extracellular membrane vesicles through blebbing of the outer membrane or cell lysis [24,25,26]. These nano-sized, lipid bilayer-enclosed particles contain LPS along with lipoproteins carrying a specific export signal [27] as well as a diverse set of periplasmic hydrolases and metabolites [28,29]. Functionally, *Bacteroidales* membrane vesicles act as extracellular organelles, extending the metabolic reach of the parent cell by degrading glycans and bile salts in the local environment [27,30]. They mediate host-microbe interactions, eliciting anti-inflammatory effects through interaction with pattern recognition receptors [31] and influencing gut microbiota composition [32]. A small fraction of these vesicles can traverse the intestinal barrier and enter the circulatory or lymphatic systems [33,34].

Here, we propose strain ASD2025^T^ as the type strain of a novel species, *Parabacteroides vesiculifaciens* sp. nov., using a polyphasic taxonomic approach combining phenotypical and phylogenomic characterization, and additionally report preliminary in vitro effects of its conditioned medium on interleukin-8 (IL-8) gene expression in HT29 cells.

## 2. Results

### 2.1. Phenotypic Features

Strain ASD2025^T^ were isolated in September 2017 from the feces of a healthy 4-year-old child living in Moscow, Russia, who had not been prescribed antibiotics for six months prior to sample collection. Isolation was performed as part of an ongoing culture-based study of the human fecal microbiome [35]. The strain was present at a concentration of approximately 1 × 10^9^ colony forming units per gram (CFU/g). It could not be identified to the species level by 16S rRNA gene sequencing but was related to the family *Parabacteroides*.

**Figure 1:**
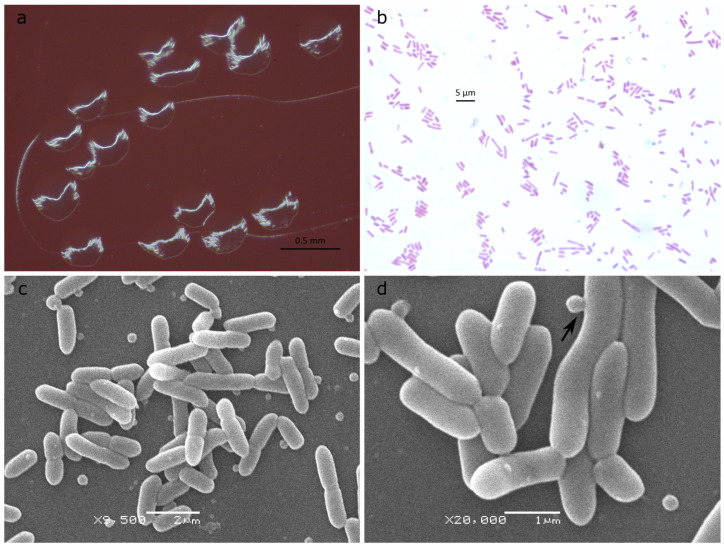
Morphology of ASD2025^T^. (**a**) Colonies on anaerobe basal agar after 48 h incubation. (**b**) Gram stain. (**c**,**d**) Scanning electron microscopy; an arrow indicates a notable example of blebbing.

Cells of strain ASD2025^T^ were obligately anaerobic, non-spore-forming, non-motile, Gram-negative polymorphic rods. Colonies on anaerobe basal agar (Oxoid Ltd., Basingstoke, UK) plates after 72 h of incubation at 37 °C under anaerobic conditions were 0.3–0.4 mm in diameter, round with irregular margin, colorless and shiny (Figure 1a). Cells in these colonies were 1.2–4.8 × 0.8 μm in size and were arranged singly (Figure 1b). Scanning electron microscopy revealed the presence of extracellular vesicles within the culture, with some observed in the process of blebbing from the outer membrane (Figure 1c,d). Isolated outer membrane vesicles were 50–90 nm in diameter according to transmission electron microscopy (Figure S1).

Strain ASD2025^T^ showed good growth at 32–43 °C, with weaker growth at room temperature (23 °C). No growth occurred at 45 °C and 47 °C. The strain grew in the presence of 0–2% NaCl, but growth was inhibited at higher concentrations. The strain tolerated 0–4% (*w*/*v*) oxgall (Sigma-Aldrich, St. Louis, MO, USA), which is equivalent to 0–40% (*w*/*v*) bile. Additionally, it successfully formed colorless colonies on bile esculin agar (HiMedia Laboratories Pvt. Limited, Mumbai, India) with 5% (*v*/*v*) defibrinated sheep blood, a medium containing 40% bile. Growth was detected at pH 6.5–8.0, and its optimum pH was 7.5. A catalase activity test was negative.

From the API 20A test results, strain ASD2025^T^ produced acid from D-glucose, D-lactose, D-sucrose, D-xylose, L-arabinose, and D-mannose, and weakly from D-raffinose, L-rhamnose and D-trehalose, but not from D-mannitol, D-maltose, salicin, glycerol, D-cellobiose, D-melezitose, or D-sorbitol. Tests for gelatin hydrolysis, aesculin hydrolysis, urease activity, and indole production were negative.

In the Rapid ID 32A identification panel, which uses chromogenic enzyme substrates, the strain ASD2025^T^ demonstrated positive reactions for only three of nine glycoside hydrolases: α-galactosidase, β-galactosidase and N-acetyl-β-glucosaminidase. It was negative for α-glucosidase, β-galactosidase 6-phosphate, α-arabinosidase, β-glucosidase, α-fucosidase and β-glucuronidase. Carbohydrate fermentation tests for mannose and raffinose within this panel were also negative. Of the 12 arylamidases tested, the strain was positive for four: leucyl glycine arylamidase, leucin arylamidase, alanine arylamidase, and glutamyl glutamic acid arylamidase; additionally, there was a weak reaction for arginine arylamidase. The alkaline phosphatase test was positive, while tests for nitrate reductase, arginine dihydrolase, and glutamate decarboxylase were negative.

**Figure 2:**
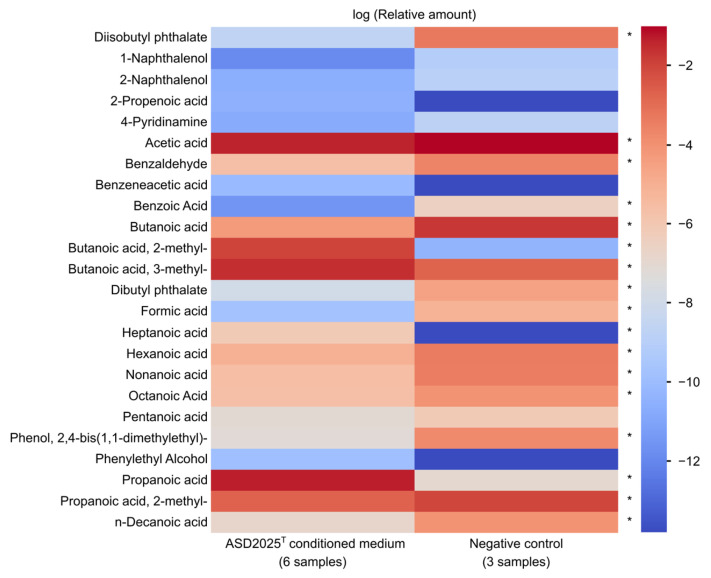
Volatile compounds in ASD2025^T^ conditioned medium. Relative concentrations in the vapor phase are used; only compounds detected in at least four samples are shown. Asterisks (*), *p* < 0.05 in Mann–Whitney test comparing two groups after Benjamini–Hochberg correction for multiple comparisons.

The major metabolic end-product of strain ASD2025^T^ grown in PYG medium was succinate. Acetate, formate, propionate, butyrate, lactate, isobutyrate, and malate were not detected by HPLC. Additionally, we assessed the produced volatile compounds using a relative non-targeted screening approach with HS-GC/MS (Figure S2). Significant increases were observed in relative amounts of 2-methylbutanoic acid and 3-methylbutanoic acid, the products of catabolism of branched-chain amino acids isoleucine and leucine, respectively (Figure 2). This method also indicated an increase in propanoic acid. In contrast, the relative amounts of 2-methylpropanoic acid (a metabolite of valine) and acetate showed a slight decrease.

The major fatty acids (>10%) of strain ASD2025^T^ were anteiso-C_15:0_ (38.2%), iso-C_17:0_ 3–OH (17.7%) and C_15:0_ (13.1%).

According to the EUCAST clinical breakpoints (v 16.0), the culture was resistant to penicillin/β-lactamase inhibitor combinations and carbapenems, but susceptible to clindamycin and metronidazole (Table S1). Additionally, we tested the isolate against several other antimicrobial agents that lack defined numerical breakpoints. This testing yielded inhibition zone diameters of <10 mm for ceftazidime, amikacin, gentamicin, vancomycin, and azithromycin; 22 mm for doxycycline hydrochloride; and 24 mm for levofloxacin.

### 2.2. General Genome Characterization

**Figure 3:**
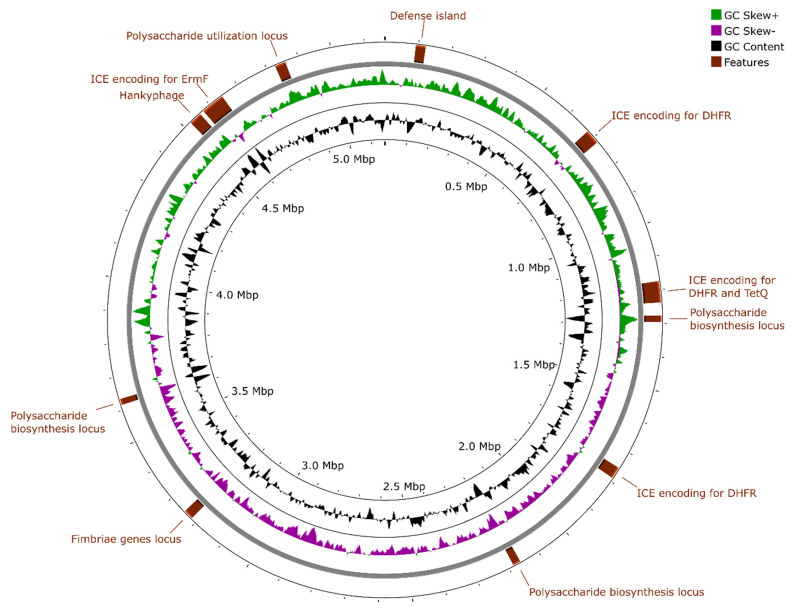
Circular map of ASD2025^T^. Rings from inner to outer represent GC-content, GC-skew and the features discussed in the text. ICE, integrative conjugative element; DHFR, dihydrofolate reductase.

The complete circular genome sequence of strain ASD2025^T^ was deposited in the GenBank nucleotide database under accession number CP187555. The genome assembly has a length of 5,161,977 bp and a GC content of 46.2% (Figure 3). The nucleotide distribution across the two strands revealed a standard genome organization composed of two halves, which are distinguished both by GC skew and the predominant direction of protein-coding regions, reflecting the movement directions of replication forks. This indicates the correctness of the assembly and the absence of recent inversions or translocations between distant genomic regions. According to CheckM v1.2.3 calculation provided by the NCBI, the assembly is 97.65% complete and has 1.63% duplicated marker genes. It contains 3979 intact coding sequences and 72 pseudogenes. No CRISPR-Cas systems were found.

The genome harbors six ribosomal RNA operons. The 16S rRNA gene sequences were 99.3–100% identical to each other, and we selected the identical ACPYIV_02535 and ACPYIV_00300 as references. The closest related sequences in NCBI rRNA_typestrains/16S_ribosomal_RNA database belonged to *P. johnsonii* M-165^T^ and *P. merdae* JCM 9497^T^ (blastn identity 96.51%), and the search against cultured type strains in NCBI refseq_genomes additionally revealed *P. hominis* NSJ-79^T^ (blastn identity 97.05%).

There are four integrative conjugative elements in the genome. All of them contain *tra* operons and encode for DNA topoisomerase (sharing 54–99% amino acid identity with Exc protein of CTnDOT; accession AJ431573), MobAB mobilization proteins as well as ParA-family ATPases involved in partitioning. All of them also possess one or more Group II intron-encoded reverse transcriptases. However, each harbor distinct antibiotic resistance genes:

- ACPYIV_02650–ACPYIV_02905: encodes a dihydrofolate reductase (ACPYIV_02845), possibly involved in trimethoprim resistance.
- ACPYIV_04610–ACPYIV_04905: encodes a dihydrofolate reductase (ACPYIV_04680) and a ribosomal protection protein TetQ (ACPYIV_04665) participating in resistance to tetracyclines.
- ACPYIV_07150–ACPYIV_07330: encodes a dihydrofolate reductase (ACPYIV_07195).
- ACPYIV_18210–ACPYIV_18590: encodes ErmF protein (ACPYIV_18520), which confers resistance to macrolides, lincosamides, and streptogramins via 23S rRNA methylation.

Notably, although phenotypic testing showed the strain was resistant to multiple beta-lactam antibiotics, no known beta-lactamase genes or other recognized resistance determinants were identified in its genome.

A putative integrative mobilizable element (ACPYIV_00405-ACPYIV_00535) is a putative defense island encoding for putative Mrr-like modification-dependent restriction endonuclease (ACPYIV_00515) and type I restriction-modification system (ACPYIV_00450- ACPYIV_00470). All these aforementioned integrative elements are not exclusive to representatives of the *Parabacteroides* genus. Homologs showing >70% coverage and >98% identity are also present in *Bacteroides* and other *Bacteroidales* genomes.

Three notable loci with low GC-content (ACPYIV_05070-ACPYIV_05155, ACPYIV_08800-ACPYIV_08905, and ACPYIV_14495-ACPYIV_14600) encode multiple genes involved in polysaccharide chain biosynthesis, which can play strain-specific roles in host interaction, immune evasion, and niche adaptation within the gut environment. Another locus (ACPYIV_19315-ACPYIV_19405) is dedicated to polysaccharide utilization and encodes for putative unsaturated glucuronyl hydrolase (ACPYIV_19365) alongside unsaturated sugar-generating polysaccharide lyase (ACPYIV_19355) together with other hydrolases and outer membrane SusCD-like complexes. A putative host-adhesion mobile element (ACPYIV_13040-ACPYIV_13140) encodes for fimbriae related to Mfa1 of *Porphyromonas gingivalis*.

**Figure 4:**
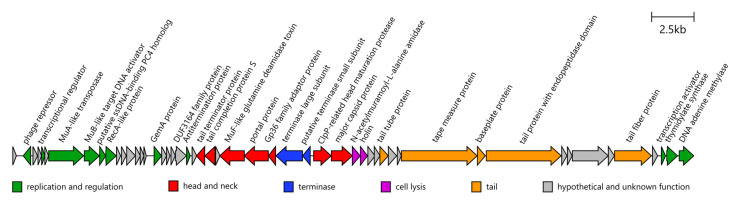
Genome map of the prophage. Each arrow represents one coding sequence.

A single prophage (ACPYIV_17920-ACPYIV_18175) is located adjacent to an ErmF-containing island. This ~40 kb region shares 59.2% nucleotide identity with *Bacteroides* phage p00 (BK010646), a member of a widespread “hankyphage” clade of temperate siphoviruses infecting bacteria of the *Bacteroidaceae* family [36,37,38]. Fragments of this genome (including genes encoding for head maturation protease, ACPYIV_18075, and tape measure protein ACPYIV_18120) share >99% identity with the related uncultured phage ctoHk1 (BK034365). These phages are known to replicate by transposition [36]; thus, the insertion site is presumed to be non-specific. The prophage apparently lacks a diversity-generating retroelement encoding a reverse transcriptase, a feature commonly reported among hankyphage-related phages, despite conservation of the other core genes (Figure 4) [37,38].

Similarly to other *Bacteroidetes*, the genome of ASD2025^T^ lacks the lpxM gene required to produce pro-inflammatory hexa-acylated LPS [16,17]. The genome also encodes for a PorA homolog (ACPYIV_08490), a putative 2-keto acid:ferredoxin oxidoreductase required for catabolism of branched-chain amino acids in other *Parabacteroides* [22,23]. Two Ntn hydrolase family proteins (ACPYIV_03060 and ACPYIV_06530) may participate in bile salt hydrolysis.

### 2.3. Phylogenomic Inference

**Figure 5:**
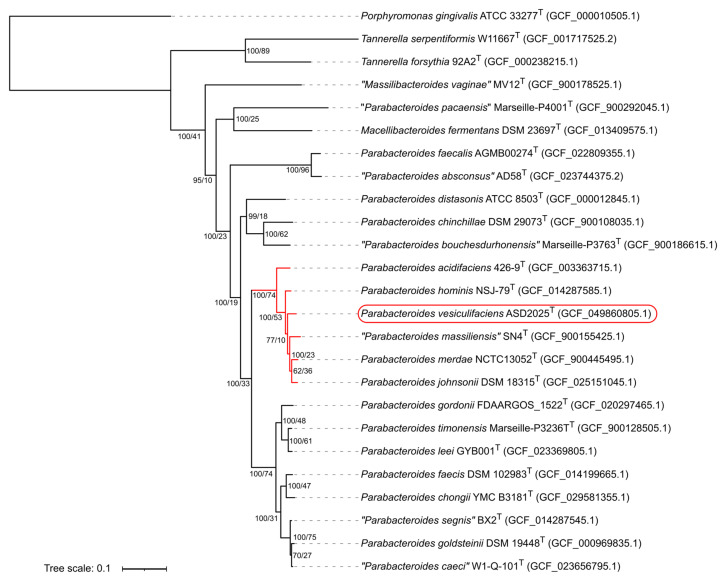
Maximum-likelihood phylogenomic tree of concatenated protein sequences inferred using edge-proportional partitional model. NCBI Refseq assembly accession numbers are provided in parentheses next to a strain name. Numbers at nodes indicate branch support calculated via partition-resampling bootstrap iterations (left value) and gene concordance factor (gCF, right value). The described strain ASD2025^T^ is marked by a red bracket on the right, and branches belonging to the “*Parabacteroides merdae* species complex” (PMSC) are highlighted in red.

Tree reconstruction based on 543 universally present proteins confirmed that the strain ASD2025^T^ is the member of the genus *Parabacteroides* (Figure 5). It is placed within a robustly inferred species complex supported by both concatenated sequences and individual protein trees. This clade includes closely related species *P. acidifaciens*, *P. hominis*, “*P. massiliensis*”, *P. merdae* and *P. johnsonii*, and we designate it as “*P. merdae* species complex” (PMSC). Calculation of gCF and sCF revealed that 73.66% of decisive individual protein trees and 52.19% of decisive alignment sites, respectively, were concordant with the existence of this branch.

There was moderate support for the grouping of “*P. pacaensis*” with *Macellibacteroides fermentans* outside the major *Parabacteroides* clade (gCF 25.48%, sCF 37.96%).

Minimal standards for the use of genome data in the taxonomy of prokaryotes require the calculation of overall genomic relatedness indices to delineate species [39]. We calculated the average nucleotide identity (ANI) between strain ASD2025^T^ and all members of the PMSC as well as *P. distasonis*, the type species for the genus (Table 1). All values were below 90%, which is well below the universally accepted species delineation threshold of 95–96% [39,40]. This demonstrates that ASD2025^T^ represents a novel, previously undescribed species.

**Figure 6:**
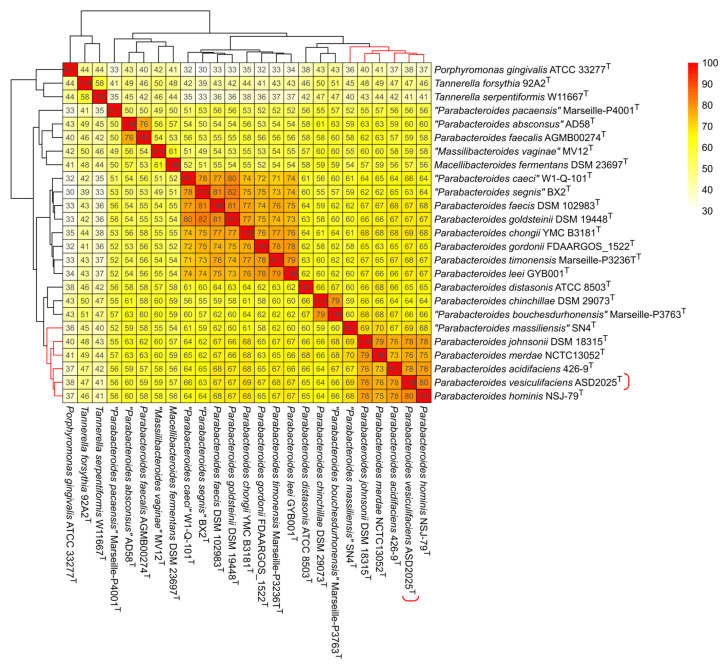
Heatmap of percentage of common proteins. Clustering of rows and columns was performed using Euclidean distances and complete linkage method. The described strain ASD2025^T^ is marked by a red bracket on the right, and branches belonging to the “*Parabacteroides merdae* species complex” (PMSC) are highlighted in red.

Although a 50% percentage of common proteins (POCP) threshold was proposed in 2014 as a universal standard for genus delineation, subsequent studies revealed that this metric has a high standard deviation and is strongly influenced by genome size [39,41]. Therefore, its utility as a general criterion is questionable. Nevertheless, it can be used as a complementary measure for phylogenomic analysis, as it is only weakly influenced by sequences of universal proteins. In this study, POCP analysis confirms that the strain ASD2025^T^ belongs to the PMSC clade (Figure 6). It shares 78–80% POCP with the type strains of *P. acidifaciens*, *P. hominis*, *P. merdae* and *P. johnsonii*. “*P. massiliensis*” has the lowest POCP values within PMSC, sharing only 69% with ASD2025^T^, which may indicate a difference in ecological niche leading to reduced gene flow with other members of the species complex. Generally, the reconstructed matrix of gene content similarity corroborates the main features of the phylogenomic tree, including relatedness of *P. faecalis* with *P. absconsus* as well as *P. chinchillae* with *P. bouchesdurhonensis*. However, it does not support the grouping “*P. pacaensis*” with *Macellibacteroides fermentans*; instead, the genera *Parabacteroides*, *Massilibacteroides* and *Macellibacteroides* formed a common cluster.

### 2.4. Glycan Degradation Proteins

**Figure 7:**
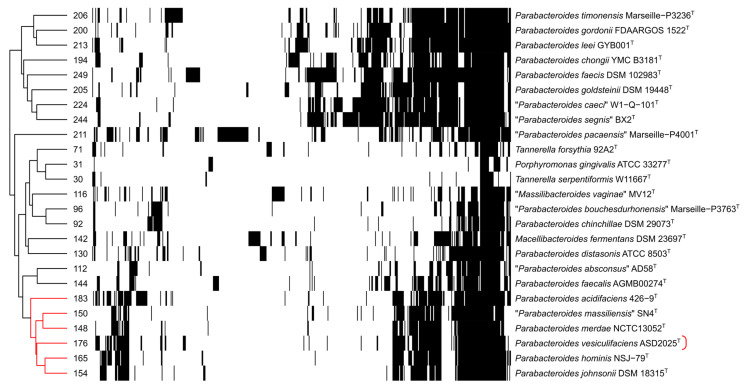
Glycan degradation protein subfamilies in *Parabacteroides* and related genera. Each column corresponds to one of 555 subfamilies; black cells indicate the presence of a subfamily in a genome, while white cells indicate absence. Rows and columns were hierarchically clustered using Euclidean distance. The total number of glycan degradation subfamilies per genome is listed on the left. The described strain ASD2025^T^ is marked by a red bracket on the right, and branches belonging to the “*Parabacteroides merdae* species complex” (PMSC) are highlighted in red.

Using run_dbcan, we identified 259 carbohydrate-active enzymes (CAZymes) in the genome of ASD2025^T^, accounting for 6.4% of its total encoded proteins. To assess the repertoire dedicated to glycan degradation, we excluded enzymes targeting peptidoglycan as well as glycosyltransferases. As a result, we identified 176 distinct CAZyme subfamilies encoded in ASD2025^T^, comprising 124 glycoside hydrolases, 32 carbohydrate-binding modules, 11 carbohydrate esterases, and nine polysaccharide lyases. Similar repertoire size is found among other species of PMSC (148–183 subfamilies), while larger variability is seen outside of it. In contrast, analyzed species of *Tannerella* harbored a drastically reduced repertoire of glycan degradation proteins (only 30–71 subfamilies). Hierarchical clustering grouped the PMSC genomes, reflecting their similar glycan utilization potential (Figure 7; see also Figure S3 for version with subfamily labels).

### 2.5. Prevalence in Metagenomes and Host Association

A search in the NCBI core_nt and WGS databases limited by the family *Tannerellaceae* revealed that the strain ASD2025^T^ has one cultured relative belonging to the same species: *Tannerellaceae* bacterium 33-180 (NCBI accession GCA_041225035.1), isolated from a specific pathogen-free mouse. The average nucleotide identity (ANI) between ASD2025^T^ and 33-180 was 99.13%.

**Figure 8:**
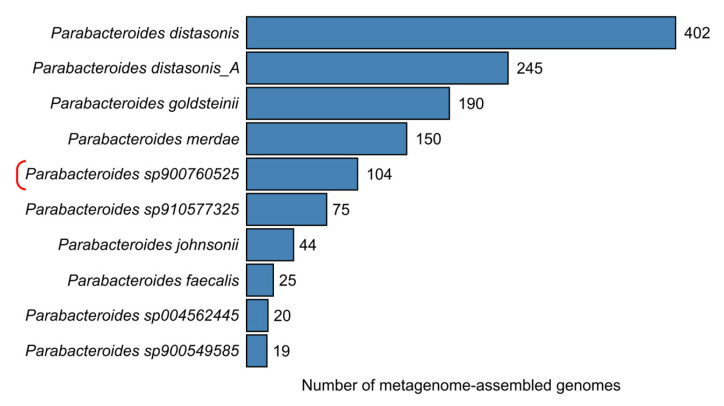
Number of metagenome-assembled genomes (MAGs) in the top 10 species-level clades of *Parabacteroides* in the GTDB database. ASD2025^T^ belongs to the clade marked with a bracket.

In the genome-based taxonomy GTDB (version 226, released 16 April 2025), these strains correspond to the species-level clade *Parabacteroides* sp900760525. This clade also includes 104 metagenome-assembled genomes (MAGs), making it the fifth most represented species within the genus *Parabacteroides* (Figure 8). However, the ecological distribution is highly skewed: only four of them are derived from human gut microbiota, while all the other 100 are from murine samples. 97 of them were assembled for the Mouse Gastrointestinal Bacteria Catalog. The marked predominance of mouse-derived genomes in this clade suggests preferential detection in murine datasets, although this observation should be interpreted cautiously because public genome collections may be affected by sampling bias.

### 2.6. Inflammatory Response to the Conditioned Medium

Since ASD2025^T^ strain was isolated from the gut microbiota, we evaluated the effect of its conditioned medium on HT29 cells, a well-established human colorectal adenocarcinoma cell line. The cells were maintained in a conditioned medium in which the strain ASD2025^T^ had been incubated. For comparison, we used the *Escherichia coli* Crooks ATCC 8739 and *P. merdae* EBA4-19 strains [42]. To test the antagonistic effect through receptor competition, we also treated the cells with *E. coli* LPS following exposure to the conditioned media and observed its impact on gene expression.

**Figure 9:**
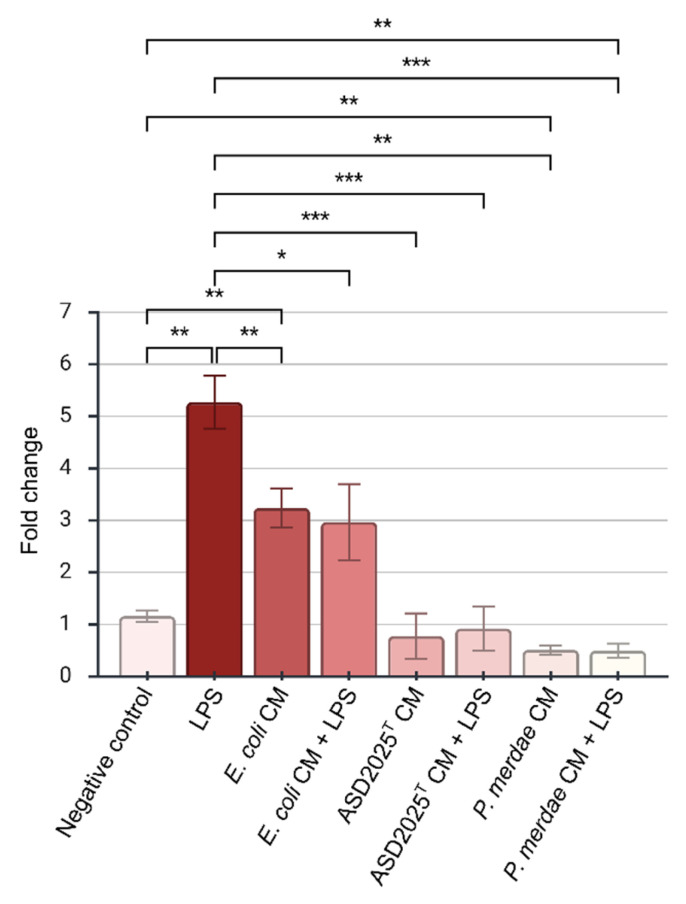
IL-8 gene expression changes in response to conditioned media (CM) by ASD2025^T^, *Parabacteroides merdae* EBA4-19, or *Escherichia coli* Crooks ATCC 8739 strains in HT29 cell line. Asterisks (*), *p* < 0.05, (**), *p* < 0.01, (***), *p* < 0.001 in Welch’s one-way ANOVA with Games–Howell multiple comparisons test against negative control (HT29 cells treated by DMEM only) and HT29 cells treated by LPS.

Whereas the *E. coli* conditioned medium caused a 2.70-fold increase in IL-8 gene expression (*p* = 0.002), the conditioned media from strains ASD2025^T^ and EBA4-19 led to a reduction in its baseline level by 1.49-fold (*p* = 0.698) and 2.27-fold (*p* = 0.002), respectively. In the presence of LPS, not an enhancement but, conversely, a reduction in IL-8 gene expression was observed, both in the case of the conditioned media from ASD2025^T^ strain (5.70-fold reduction, *p* = 0.0002) and the EBA4-19 strain (10.60-fold reduction, *p* = 0.001). Thus, a clear suppressive effect on the IL-8 response to *E. coli* LPS was observed for the conditioned media from strains ASD2025^T^ and EBA4-19 under the tested conditions (Figure 9).

## 3. Discussion

The characterization of strain ASD2025^T^ expands the known diversity of the genus *Parabacteroides*, a group of gut commensals increasingly recognized for their role in host metabolism and immune system interactions.

Our polyphasic approach establishes this isolate as a novel species, for which we propose the name *Parabacteroides vesiculifaciens* sp. nov. This taxonomic decision is based on 16S rRNA gene identity (97.05% and lower) and average nucleotide identity (89.9% and lower) with previously described type species, both below universally accepted thresholds for species delineation [39,40,43], as well as the position and branch length in the phylogenomic tree.

The prevalence of the anteiso-C_15:0_ among the long chain fatty acids is characteristic of the *Parabacteroides* species [2,11]. On the other hand, the large amount of produced straight-chain C_15:0_ is characteristic of the related genus *Macellibacteroides* [44,45,46]. The production of succinate as a major metabolic product is also widespread [2], but not the universal [47] trait of the genus *Parabacteroides*. The identified branched short chain fatty acids, originating from amino acid catabolism, are also typical for *Parabacteroides*, suggesting shared core metabolism and potential functional similarities in host interaction [2,22,23,48].

Three independent approaches—phylogenomic inference based on core protein sequences, percentage of common proteins, and encoded subfamilies of glycan degradation proteins—collectively indicate the existence of a “*P. merdae* species complex” (PMSC). This monophyletic group with putatively similar metabolic capacities, besides proposed species *P. vesiculifaciens*, includes *P. acidifaciens*, *P. hominis*, “*P. massiliensis*”, *P. merdae*, and *P. johnsonii*. However, shared saccharolytic activities do not imply a similar ecological niche: while *P. merdae* is widespread in human microbiota [6,7], *P. vesiculifaciens* is putatively more associated with mice; isolation of ASD2025^T^ from a human sample may reflect transient colonization or a rare human-associated sub-lineage.

The API biochemical tests show that ASD2025^T^ possesses a comparatively narrow enzymatic repertoire: it is negative for many enzymes, especially arylamidases, that are widespread in related *Parabacteroides* species [2,47,48], hinting at low proteolytic capability. However, its high number of encoded glycan degradation proteins, comparable to related PMSC species, suggests a highly saccharolytic metabolism, in stark contrast to the type genus of the family, *Tannerella*. Notably, strain ASD2025^T^ possesses a more extensive carbohydrate degradation repertoire than *P. distasonis*, the most widespread *Parabacteroides* species in the human gut, which was previously described as having limited genetic potential for polysaccharide degradation [49]. However, while *P. distasonis* was described to be enriched in glycoside hydrolase family 13 (GH13), predominantly associated with α-amylase activity [49], ASD2025^T^ harbors a smaller amount of these enzymes than *P. distasonis* ATCC 8503 (two versus eight).

An additional point requiring consideration is the discrepancy between the observed resistance to several β-lactams and the absence of known β-lactamase genes, unlike in resistant clinical isolatations of *Bacteroides fragilis* [50]. The resistance may reflect not only acquired β-lactamases but also intrinsic mechanisms, including reduced permeability, efflux, altered target, or determinants that remain poorly represented in current annotation databases. Therefore, the genomic basis of the observed phenotype in ASD2025ᵀ remains unresolved, as well as the possibility of horizontal transfer to other species.

The absence of the *lpxM* gene suggests the production of hypo-acylated lipid A with anti-inflammatory properties, similar to other *Bacteroidales* [18,19]. This genetic feature likely explains the anti-inflammatory potential observed in vitro, where conditioned medium of ASD2025^T^ antagonized the pro-inflammatory effect of *E. coli* LPS on HT29 cells, similarly to related PMSC species *P. merdae*. The similar results were obtained previously for crude membrane fractions of *P. distasonis* as well as autoclaved *P. goldsteinii* cells and purified *P. goldsteinii* LPS [17,19,51]. However, the obtained result may not be generalizable over all *Parabacteroides* species, as the same assay as used in this study detected pro-inflammatory activity without significant receptor masking in *P. absconsus* [6]. The observation of abundant vesicle-like structures by scanning electron microscopy and transmission electron microscopy provides a plausible mechanism of secretion of LPS and other envelope-associated components without cell lysis in ASD2025^T^.

## 4. Materials and Methods

### 4.1. Isolation and Preservation

Growth of the strain was first discovered on anaerobe basal agar (Oxoid Ltd., Basingstoke, UK) supplemented with 5% (*v*/*v*) defibrinated sheep blood (EcoLab, Moscow, Russia) and incubated under anaerobic conditions at 37 °C for 72 h.

Upon isolation, the strain was cultured anaerobically in anaerobe basal agar (Oxoid, UK) and supplemented with 5% (*v*/*v*) defibrinated sheep blood. Cultures were incubated at 37 °C for 48–96 h in an atmosphere of 85% N_2_/10% H_2_/5% CO_2_ in anaerobic jars (Schuett-Biotec, Göttingen, Germany) with catalysts containing palladium-coated pellets. Strains were preserved by freeze-drying of bacterial suspensions in 10% (*w*/*v*) sucrose and 1% (*w*/*v*) gelatin solution.

### 4.2. Phenotypical and Chemotaxonomic Characterizations

Cell morphology was examined after 72 h incubation on anaerobe basal agar (Oxoid Ltd., Basingstoke, UK) with 5% (*v*/*v*) defibrinated sheep blood using Gram staining and bright-field microscopy (Axio Scope. A1; Carl Zeiss, Oberkochen, Germany).

For scanning electron microscopy (SEM), 48 h bacterial cultures in Schaedler anaerobe broth (Oxoid, UK) were diluted 1:1 (*v*/*v*) with phosphate-buffered saline (PBS; pH 7.4; PanReac AppliChem, Darmstadt, Germany) supplemented with 5% (*w*/*v*) sucrose to mitigate osmotic shock during partial air-drying. Aliquots (10–20 µL) were dispensed onto clean glass coverslips. After ~30 min at room temperature to allow partial drying and adhesion, samples were fixed in 2.5% glutaraldehyde (Electron Microscopy Sciences, Hatfield, PA, USA) prepared in PBS with 5% sucrose for 15 min. Coverslips were then dehydrated in graded ethanol (30%, 50%, 70%, 80%, 95% for 15 min each), followed twice by 100% ethanol for 10 min. The solvent was replaced with acetone (2 × 10 min). Samples were mounted on SEM stubs using carbon adhesive tabs and sputter-coated with a thin gold layer in argon plasma. Imaging was performed on a scanning electron microscope JSM-6380 (JEOL Ltd., Tokyo, Japan) operated at 20 kV under standard conditions.

To isolate outer membrane vesicles, about 200 mL of a 24 h liquid bacterial culture was centrifuged at 4500× *g* at 4 °C. To remove residual cells, the supernatant was filtered through a 0.45 µm porous membrane. The filtrate was subjected to ultracentrifugation at 100,000× *g* for 2 h (Optima L-90K ultracentrifuge; Beckman Coulter, Brea, CA, USA). The supernatant was discarded, and the pellet was washed with sterile phosphate-buffered saline (PBS) and filtered through a sterile 0.2-μm pore polyvinylidene difluoride (PVDF) membrane (Merck Millipore, Burlington, MA, USA). The ultracentrifugation procedure was repeated two times. The vesicle pellet was resuspended in distilled water or 150 mM NaCl (pH 6.5). The concentration of OMVs was quantified as the dry precipitate. For 3 min, 5 µL of sample were negatively stained with 2% (wt/vol) uranyl acetate. Images were obtained using a JEM-1400 (Jeol, Tokyo, Japan) transmission electron microscope equipped with a Rio-9 camera (Gatan Inc., Pleasanton, CA, USA) at 120 kV.

Cellular long-chain fatty acid profiles were determined from 96 h cultures grown in PYG medium (peptone, 1.25 g/L, yeast extract powder 1.25 g/L, glucose 3.0 g/L). Fatty acids were extracted from dry biomass samples via acid methanolysis using 0.4 mL of 1.2 M HCl in methanol at 80 °C for 45 min. The resulting fatty acid methyl esters were extracted with hexane and converted to trimethylsilyl derivatives. Separation and analysis were performed using a Agilent 6850-5973 GC-MS (Agilent Technologies, Santa Clara, CA USA) in a 5% phenyl-95%-methyl-polysiloxane column 25 × 0.25 mm with a phase thickness of 0.25 μm (Rxi-5ms, Restek, Bellefonte, PA, USA). The temperature gradient was programmed from 135 °C to 320 °C at a rate of 7 °C min^−1^.

Products of PYG medium fermentation were assayed with an HPLC system (Knauer GmbH, Berlin, Germany). The analytical column was Inertsil ODS-3 (5 µm, 250 × 4.6 mm; Dr. Maisch GmbH, Ammerbuch, Germany). Chromatography was carried out in 20 mM H_3_PO_4_ at 210 nm, at a temperature of 35 °C and a pressure of 130 bar, resulting in the eluent flow rate of 1.0 mL per min. The products were identified using standard solutions of acids 2 g L^−1^ (Sigma-Aldrich, St. Louis, MO, USA) according to a retention time. Formed compound concentrations were calculated from height and peak area using EuroChom software, v. 3.05 P5 (Knauer GmbH, Berlin, Germany).

For HS-GC/MS, 1 mL of samples (conditioned medium of PYG as negative control) were placed into 10 mL screw-cap vials for a HS-20 headspace extractor (Shimadzu, Kyoto, Japan). A mixture of 0.15 g of salts (ammonium sulfate and potassium dihydrogen phosphate in a ratio of 4:1) was added to increase solution ionic strength. Headspace extractor settings used: oven temperature 80 °C, sample line temperature 220 °C, transfer line temperature 220 °C, equilibrating time 15 min, pressurizing time 2 min, load time 0.5 min, injection time 1 min, needle flush time 7 min. The vials were sealed and analyzed on a Shimadzu QP2010 Ultra GC/MS with a Shimadzu HS-20 headspace extractor, a VF-WAXMS column with a length of 30 m, a diameter of 0.25 mm, and a phase thickness of 0.25 microns. Initial column temperature 80 °C, heating rate 20 °C/min to 240 °C, exposure 20 min. Carrier gas—helium 99.9999, injection mode—splitless, flow rate 1 mL/min. Ion source temperature—230 °C. Interface temperature 240 °C. Total ionic current (TIC) monitoring mode was used. To analyze the obtained mass spectra, the NIST 2014 mass spectra library with automated mass spectral deconvolution and identification system (AMDIS version 2.72) was used. Only compounds detected in at least four of nine samples were used. Peak areas computed by AMDIS for the selected compounds were recalculated as a percentage of the total reliably identified compounds. Comparison of relative amounts was made using the Mann–Whitney test with Benjamini–Hochberg correction for multiple comparisons.

Biochemical reactions were determined in triplicate by using the API 20A anaerobe test kit (bioMérieux, Marcy l’Etoile, France) and Rapid ID 32A anaerobe identification kit (bioMérieux, Marcy l’Etoile, France) using incubation for 48 h and 4 h, respectively. Catalase activity was determined by adding 3% H_2_O_2_ solution to fresh cells. The effects of UKon growth were examined on anaerobe basal agar (Oxoid Ltd., Basingstoke, UK) with 5% (*v*/*v*) defibrinated sheep blood by culturing at 23, 32, 37, 42, 43, 45, and 47 °C for 72 h. In addition, the same medium was supplemented with 0–4% (*w*/*v*) oxgall (Sigma-Aldrich, St. Louis, MO, USA) to test the susceptibility of the strain to bile, together with bile esculin agar (HiMedia Laboratories Pvt. Limited, Mumbai, India) with 5% (*v*/*v*) defibrinated sheep blood. Susceptibility to salinity was checked in Schaedler anaerobe broth (Oxoid Ltd., Basingstoke, UK) with addition of 0–4% (*w*/*v*) NaCl. Disk diffusion tests were performed using Oxoid susceptibility testing disks.

Effect of pH on growth was tested using the broth of the following composition: tryptone soya broth, 10.0 g/L; peptone, 5.0 g/L; yeast extract powder, 5.0 g/L; glucose, 5.0 g/L; and haemin, 0.01 g/L, adjusted to pH 5.5–10.5 (with 0.5 unit step changes). The buffer systems were: pH 4.0–6.0, 0.1 M citric acid–0.1 M sodium citrate; pH 6.5–8.0, 0.1 M KH_2_PO_4_—0.1 M NaOH; and pH 8.5–10.5, 0.1 M Na_2_CO_3_ x10H_2_O—0.1 M NaHCO_3_. Inoculated tubes were incubated at 37 °C for 72 h.

Prior to antimicrobial susceptibility testing, isolates were cultured on anaerobe basal agar (Oxoid Ltd., Basingstoke, UK) supplemented with 5% (*v*/*v*) defibrinated sheep blood and incubated in an atmosphere of 85% N_2_, 10% H_2_, and 5% CO_2_ at 37 °C for 24 h in anaerobic jars (Schuett-Biotec, Göttingen, Germany) containing catalysts with palladium-coated pellets. The isolates were tested using a disk diffusion procedure according to the European Committee on Antimicrobial Susceptibility Testing (EUCAST) recommendations [52,53]. From the plates, a bacterial suspension was prepared in 0.9% saline to a density of a 1.0 McFarland standard and applied to Fastidious Anaerobe Agar (NEOGEN, Lansing, MI, USA) supplemented with 5% defibrinated horse blood. Concentrations of antibiotics are provided in Table S1. Imipenem and meropenem disks were obtained from Bio-Rad (Bio-Rad, Marnes-la-Coquette, France), ertapenem disks from BD BBL (Becton Dickinson Franklin Lakes, NJ, USA), and all other disks were obtained from Bioanalyse (Bioanalyse Limited, Yenimahalle-Ankara, Türkiye). The plates were incubated at 37 °C in an anaerobic atmosphere, and results were read after 17 h. *Bacteroides fragilis* ATCC 25285 was included as a quality control strain.

### 4.3. Genome Sequencing and Analysis

Genomic DNA was extracted using the QIAamp DNA Mini Kit (Qiagen, Hilden, Germany). The 16S rRNA gene fragments were Sanger sequenced in Evrogen JSC (Moscow, Riussia). For whole-genome sequencing, a hybrid approach was employed. Long-read sequencing was conducted by LLP “Genomed”. Sequencing was performed on a MinION Mk1B device (Oxford Nanopore Technologies, Oxford, UK) with a FLO-MIN114 (R10.4.1) flow cell, using real-time SUP basecalling with Dorado 7.2.13 (Oxford Nanopore Technologies, Oxford, UK). Short-read sequencing was carried out in the Lopukhin Federal Research and Clinical Center of Physical-Chemical Medicine of the Federal Medical Biological Agency using DNBSEQ-G400 (BGI, Shenzhen, China).

A circular genome sequence was assembled from long reads using Flye v2.9.3 [54]. The assembly was subsequently polished with short reads using pypolca v0.3.1 and Polypolish v0.6.0 [55,56]. The final sequence was annotated using the NCBI Prokaryotic Genome Annotation Pipeline (PGAP) v6.10 [57]. Completeness and contamination were assessed with CheckM v1.2.3 [58]. Average nucleotide identities were calculated with PyANI-plus using the ANIm method [59]. Manual annotation of the genes of interest was performed using a combination of sequence similarity and structure-based approaches. HHpred searches were performed against the PDB70_mmCIF70_30_Mar, PfamA-v37, UniProt-SwissProt-viral70_3_Nov_2021, and NCBI_Conserved_Domains(CD)_v3.19 databases [60]. Foldseek searches [61] were performed using structures predicted by AlphaFold 3 [62]. A circular map was visualized with Proksee [63]. Antibiotic resistance genes were searched using AMRFinderPlus 4.2.5 and CARD Resistance Gene Identifier 6.0.3 [64,65]. Prophage was detected using Phigaro 2.4.0 and visualized with clinker [66,67].

For comparative genomic analysis, we selected type strains of the *Tannerellaceae* family (except *Candidatus* taxa) according to the LPSN database as well as *Porphyromonas gingivalis* ATCC 33277^T^ as an outgroup. Additionally, we added genera *Massilibacteroides* and *Macellibacteroides* as closely related according to GTDB genome-based phylogeny [13].

Genome assemblies of the selected strains were downloaded from the NCBI RefSeq database. The following species were excluded from further analysis: “*P. provencensis*” as a later heterotypic synonym of *P. chinchillae* (ANIm between strains Marseille-P3668^T^ and DSM 29073^T^ equals 98.26%), *P. chartae* as a later heterotypic synonym of *Macellibacteroides fermentans* (strains DSM 24967^T^ and DSM 23697^T^: 97.81%) [46], and *P. propionicigenes* as a later heterotypic synonym of *P. leei* (strains HA3406^T^ and GYB001^T^: 99.27%).

Orthologous gene families were identified using PIRATE 1.0.5 with a default range of amino acid identity thresholds [68]. As a result, we obtained a core proteome, comprising 543 conserved protein groups that were encoded in every genome in a single copy without any fusion/fission events. The amino acid sequences of the core proteins were aligned using MAFFT 7.310 [69]. Phylogenomic inference was performed on this set with IQ-TREE 3.0.1 using an edge-proportional partitional model with 1000 partition-resampling bootstrap iterations [70]. Gene concordance factor (gCF) and site concordance factor based on maximum likelihood (sCF), representing the percentages of decisive gene trees or alignment sites supporting each branch, were calculated for the obtained tree [71,72].

CAZyme annotation was performed using run_dbcan v. 5.2.4, a standalone version of dbCAN3. To obtain glycan-degrading enzyme subfamilies, we used the results recommended by run_dbcan, excluding enzymes with peptidoglycan as the preferred substrate. The annotations were then split into domains (e.g., “GH97_e19|CE17_e0” was counted as “GH97_e19” and “CE17_e0” separately), and glycosyltransferases were excluded as they are primarily biosynthetic enzymes. The final matrix represented the presence/absence of each CAZyme subfamily within the genome.

### 4.4. Modulating Effect of Conditioned Medium on Human Cells

The modulating effect of conditioned medium from strain ASD2025^T^ on IL-8 gene expression was investigated using a HT29 cell model. Cells were grown in DMEM (Dulbecco’s Modified Eagle Medium) High Glucose 4.5 g/L (PanEco, Moscow, Russia) supplemented with 10% (*v*/*v*) fetal bovine serum (Capricorn Scientific GmbH, Ebsdorfergrund, Germany), antibiotics penicillin (100 IU/mL) and streptomycin (100 μg/mL) (PanEco, Moscow, Russia), and 1% L-glutamine. The cultures were maintained at 37 °C under conditions of 5% CO_2_ eight days before the experiment. For the preparation of conditioned medium, strain ASD2025^T^ was grown in Schaedler anaerobe broth (Oxoid Ltd., Basingstoke, UK) at 37 °C under anaerobic, non-agitating conditions for 48 h, pelleted by centrifugation (40 min, 3500× *g*), and resuspended in the initial volume of the DMEM High Glucose 4.5 g/L medium with the addition of 20 mM HEPES (4-(2-hydroxyethyl)-1-piperazineethanesulfonic acid) (PanEco, Moscow, Russia). After centrifugation under the same conditions and removal of the supernatant, a bacterial suspension was prepared by diluting it with DMEM High Glucose 4.5 g/L with 20 mM HEPES (PanEco, Russia) to a concentration of approximately 3.0 ×  10^8^ CFU/mL (1.0 McFarland standard) using a densitometer device (McFarland Densitometer DEN-1, Biosan, Riga, Latvia). The cultures were then incubated for 20 h at 37 °C under anaerobic, non-agitating conditions. Cells were removed by centrifugation, and the pH of the supernatants was adjusted to pH 7.4 followed by 0.22 μm filter sterilization (Merck Millipore, Burlington, MA, USA). The same protocol was performed for the preparation of conditioned medium from *P. merdae* EBA4-19 [42] and *E. coli* ATCC 8739.

The experiment (Table 2) was performed in eight biological replicates. Cells were incubated with 450 μL/well of conditioned media or negative control media for 30 min, then 10 μL lipopolysaccharide (LPS) solution was added to the final concentration of 100 ng/mL with subsequent incubation for 4 h. The expression of IL-8 was determined by RT-qPCR using housekeeping gene glyceraldehyde 3-phosphate dehydrogenase (GAPDH) as a reference [73]. For the expression measurement, total RNA was extracted with QIAGEN RNeasy Plus Universal Kits (Qiagen, Hilden, Germany) according to the instructions of the manufacturer. The first strand cDNA was synthesized using a reverse transcriptase MMLV kit (Evrogen, Moscow, Russia) with a universal oligo(dT)15-primer.

Reverse transcription was performed for 1 h at 37 °C and 10 min at 70 °C to stop the reaction according to the manufacturer’s instructions. qRT-PCR was performed using the Bio-Rad CFX 96 Real-Time Detection System (Bio-Rad, Hercules, CA, USA). Oligonucleotide primers and thermocycling conditions for the amplification are shown in Table 3. qPCRmix-HS-SYBR (Evrogen, Moscow, Russia) was used to prepare the reaction mixtures. The specificity of reaction products was confirmed by melting temperature analysis (from 70 °C to 95 °C in 0.5 °C/15 s increments). The mRNA level of GAPDH was used to normalize the expression ratio of genes of interest. All reactions were carried out three times, and the final analysis was based on the mean of the reactions. The relative quantification method of 2^−ΔΔct^ was used in further calculations [73]; comparison of fold changes was made by Welch’s one-way ANOVA with Games–Howell multiple comparisons test.

## 5. Conclusions

Based on the described chemotaxonomic and genotypic properties it was concluded that the strain ASD2025^T^ represents a novel species within the genus *Parabacteroides*, for which the name *Parabacteroides vesiculifaciens* sp. nov. is proposed. The main characteristics of the novel taxon in comparison with some related species are provided in Table 4.

### Description of Parabacteroides vesiculifaciens sp. nov.

*Parabacteroides vesiculifaciens* (ve.si.cu.li.fa′ci.ens; L. fem. dim. n. vesicula, a small bladder, vesicle; L. part. adj. *faciens*, making; N.L. part. adj. *vesiculifaciens*, vesicle-making, referring to the production of extracellular vesicles).

Cells are obligately anaerobic, non-motile, mesophilic, Gram-negative polymorphic rods. Growth occurs at 32–43 °C (with weak growth at 23 °C), pH 6.5–8.0 (optimum pH 7.5), and 0–2% (*w*/*v*) NaCl. Acid is produced from D-glucose, D-lactose, D-sucrose, D-xylose, L-arabinose, and D-mannose, and weakly from D-raffinose, L-rhamnose and D-trehalose, but not from D-mannitol, D-maltose, salicin, glycerol, D-cellobiose, D-melezitose, or D-sorbitol. Tests for catalase, gelatin hydrolysis, aesculin hydrolysis, urease activity, and indole production are negative. Positive for α-galactosidase, β-galactosidase, N-acetyl-β-glucosaminidase, leucyl-glycine arylamidase, leucine arylamidase, alanine arylamidase, glutamyl-glutamic acid arylamidase, and alkaline phosphatase. Weakly positive for arginine arylamidase. Negative for α-glucosidase, β-galactosidase-6-phosphate, α-arabinofuranosidase, β-glucosidase, α-fucosidase, β-glucuronidase, nitrate reductase, arginine dihydrolase, glutamate decarboxylase, tyrosine arylamidase, phenylalanine arylamidase, glycine arylamidase, histidine arylamidase, serine arylamidase, proline arylamidase, and pyroglutamic acid arylamidase. The major fatty acids are anteiso-C_15:0_, iso-C_17:0_ 3–OH, and C_15:0_.

The type strain is ASD2025^T^ (=VKM B-3926^T^ = JCM 37967^T^), isolated from human feces. The GenBank accession number for the complete genome sequence is CP187555. The DNA G+C content of the genome of the type strain is 46.2 mol%.

## Figures and Tables

**Table 1:** Average nucleotide identity values (ANI) between ASD2025^T^ and the related type strains.

Strain	ANI, %	Query Coverage, %
*P. hominis* NSJ-79^T^	89.9	66.9
*P. johnsonii* DSM 18315^T^	89.7	66.3
*P. merdae* NCTC13052^T^	89.0	67.9
“*P. massiliensis*” SN4^T^	88.4	54.1
*P. acidifaciens* 426-9^T^	85.5	49.3
*P. distasonis* ATCC 8503^T^	83.3	5.97

**Table 2:** Scheme of inflammatory response testing.

Samples	Incubation for 30 min	Addition Before Incubation for 4 h
Negative control	DMEM high glucose with 20 mM HEPES	-
LPS	DMEM high glucose with 20 mM HEPES	LPS *E. coli* O55:B5
*E. coli*	Conditioned medium from *E. coli* ATCC 8739	-
*E. coli* + LPS	Conditioned medium from *E. coli* ATCC 8739	LPS *E. coli* O55:B5
ASD2025T	Conditioned medium from ASD2025T	-
ASD2025T + LPS	Conditioned medium from ASD2025T	LPS *E. coli* O55:B5
*P. merdae*	Conditioned medium from *P. merdae* EBA4-19	-
*P. merdae* + LPS	Conditioned medium from *P. merdae* EBA4-19	LPS *E. coli* O55:B5

**Table 3:** Oligonucleotide primers and thermocycling conditions for amplification.

Gene	Primer Sequences (5′-3′)	PCR Protocol	Reference
*GAPDH*	F-CTTTGACGCTGGGGCTGGCATT R-TTGTGCTCTTGCTGGGGCTGGT	ID—95 °C 5 min, D—96 °C 20 s, A 56 °C 30 s, E 72 °C 20 s, NC—40.	[74]
*IL-8*	F-CAAGGAAAACTGGGTGCAGA R-CCTACAACAGACCCACACAA	ID—95 °C 5 min, D—94 °C 20 s, A 60 °C 30 s, E 72 °C 20 s, NC—40.	[75]

ID—initial denaturing; NC—number of cycles; D—denaturing; A—annealing conditions; E—elongation.

**Table 4:** Differential characteristics between strain ASD2025^T^ (data from this study) and the type strains of related species: 1, *P. distasonis* JCM 5825^T^ [2]; 2, *P. merdae* JCM 9497^T^ [2]; 3, *P. johnsonii* M-165^T^ [48]; 4, *P. acidifaciens* 426-9^T^ [47].

Characteristic	ASD2025^T^	1	2	3	4
G+C content (mol%) *	46.2	45.1	45.3	45.2	45.9
Genome assembly size (Mb) *	5.16	4.81	4.40	4.75	5.10
Major cellular fatty acids (>10%)	anteiso-C_15:0_, iso-C_17:0_ 3-OH, C_15:0_	anteiso-C_15:0_, iso-C_17:0_ 3-OH C_18:1_ ω9c	anteiso-C_15:0_, iso-C_17:0_ 3-OH C_18:1_ ω9c	anteiso-C_15:0_, iso-_C17:0_ 3-OH	anteiso-C_15:0_, iso-_C17:0_ 3-OH
Catalase	-	+	-	+	-
β-Glucuronidase	-	-	+	+	+
α-Fucosidase	-	-	-	-	+
Glutamic acid decarboxylase	-	+	+	+	+
Tyrosine arylamidase	-	+	+	+	+
Phenylalanine arylamidase	-	+	+	+	+
Glycine arylamidase	-	+	+	+	+
Histidine arylamidase	-	+	+	+	+
Aesculin hydrolysis	-	+	+	+	+
Acid production from:					
L-arabinose	+	-	-	+	+
D-melezitose	-	+	-	-	-
Salicin	-	+	-	-	-
D-mannitol	-	-	-	-	+
Glycerol	-	-	-	-	+

“+”—presence of a feature; “-”—absence of a feature; * G+C content and genome size were calculated based on publicly available whole genome sequences.

## Data Availability

The microorganism was deposited into the Japan Collection of Microorganisms (JCM) under the number JCM 37967^T^ and All-Russian Collection of Microorganisms (VKM) under the number VKM B-3926^T^. The whole-genome nucleotide sequence of the strain is openly available in NCBI Genbank under accession number CP187555.

## Supplementary Materials

The following supporting information can be downloaded at: https://www.mdpi.com/article/10.3390/ijms27062763/s1.
