# Supplementary material for: Parabacteroides vesiculifaciens sp. nov., a Novel Immunomodulatory, Vesicle-Producing Gut Commensal Isolated from the Human Gut
Source: Int J Mol Sci. 2026 Mar 18;27(6):2763. doi: 10.3390/ijms27062763 (PMC13026095; doi:10.3390/ijms27062763)
Supplement: Supplementary file 1 [file ijms-27-02763-s001.zip › ijms-4187136-supplementary.pdf]

International Journal of
Molecular Sciences

Article

Supplementary Materials

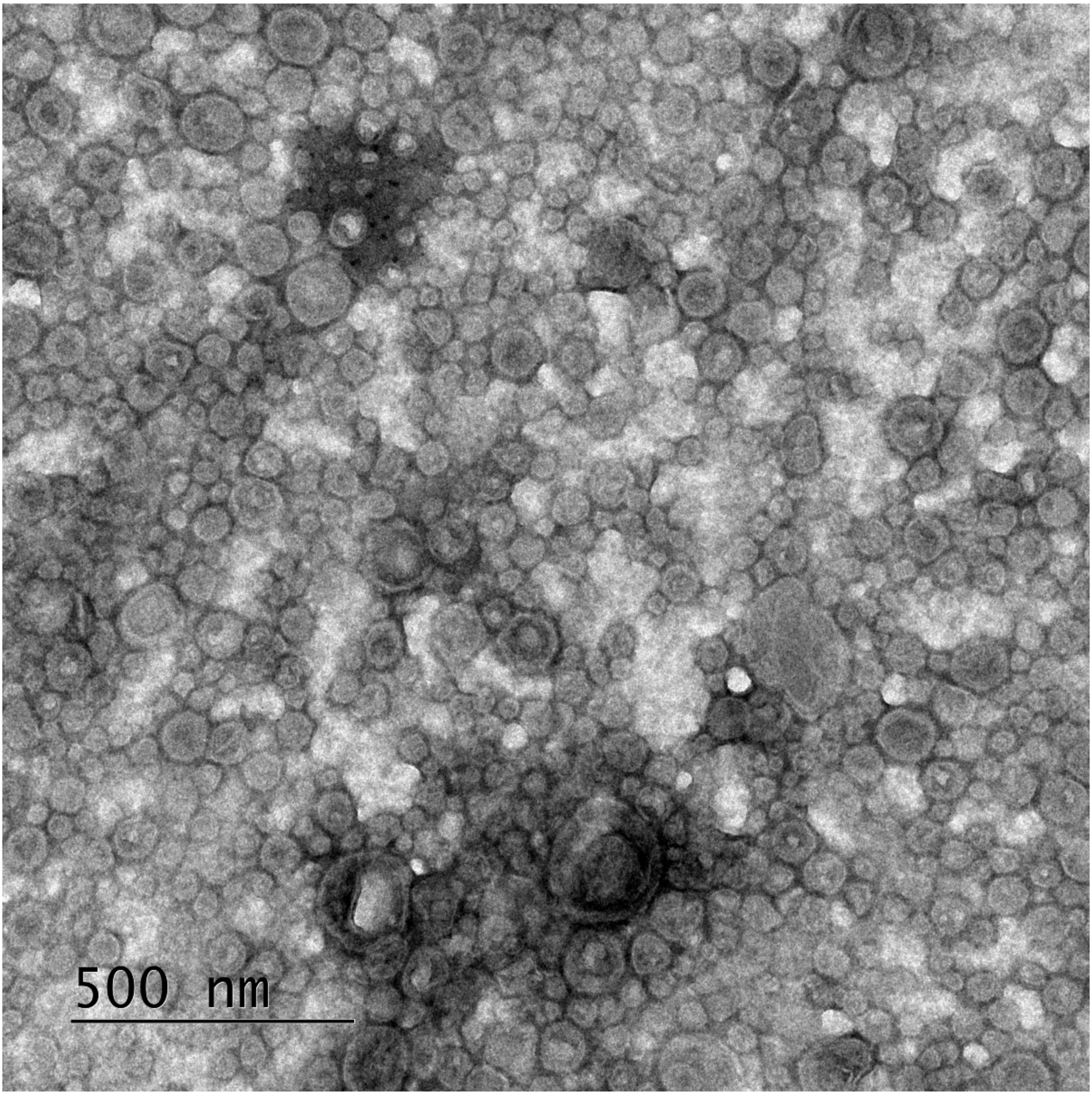

Figure S1. Transmission electron microscopy of outer membrane vesicles of the strain ASD2025^{T}.

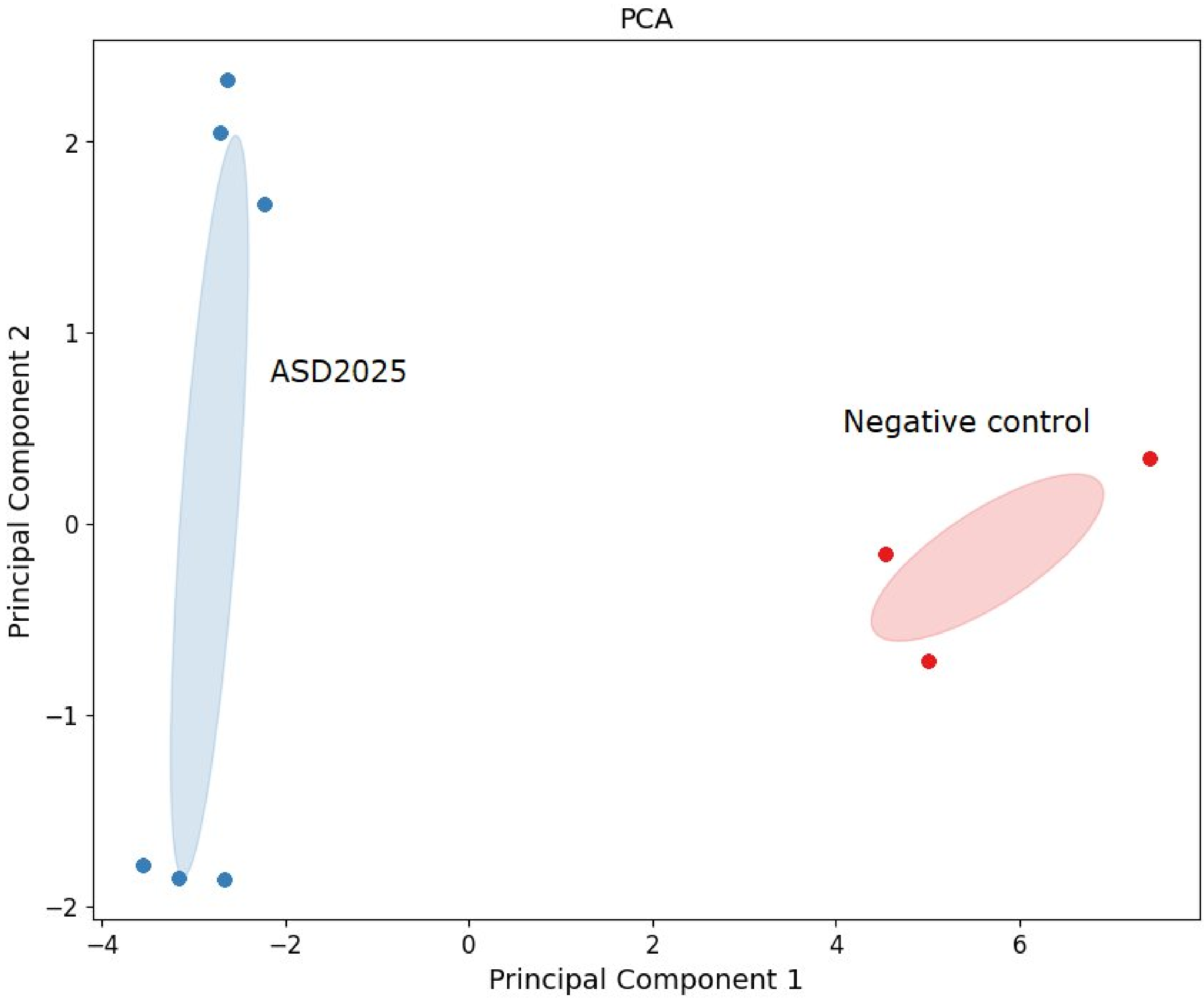

Figure S2. Principal component analysis of the total HS-GC/MS dataset of volatile compound relative concentrations in the vapor phase obtained from bacterial culture media.

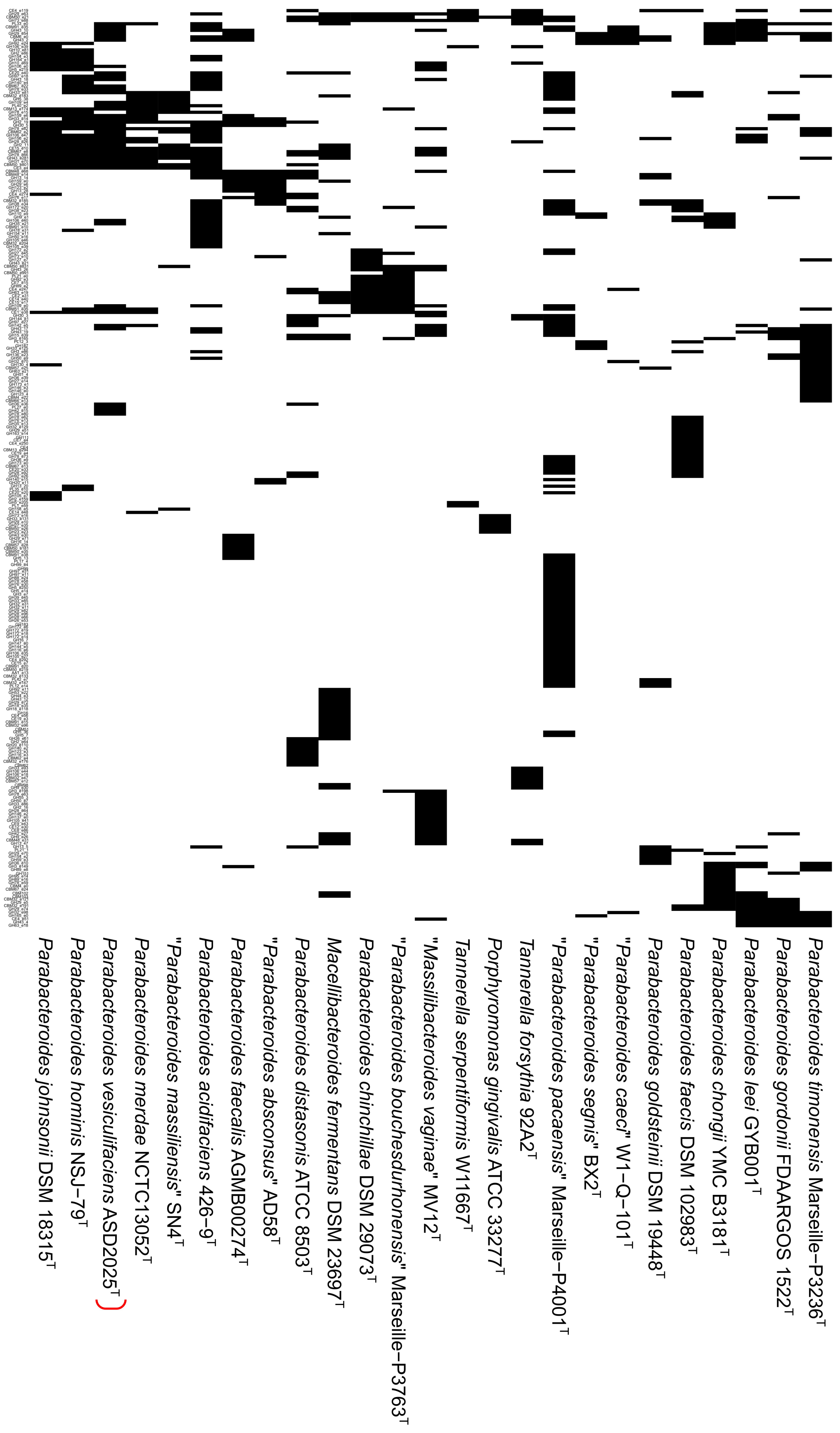

Figure S3 (part 1). Glycan degradation protein subfamilies in *Parabacteroides* and related genera. Each row corresponds to one of 555 subfamilies; black cells indicate the presence of a subfamily in a genome, while white cells indicate absence. Rows and columns were hierarchically clustered using Euclidean distance.

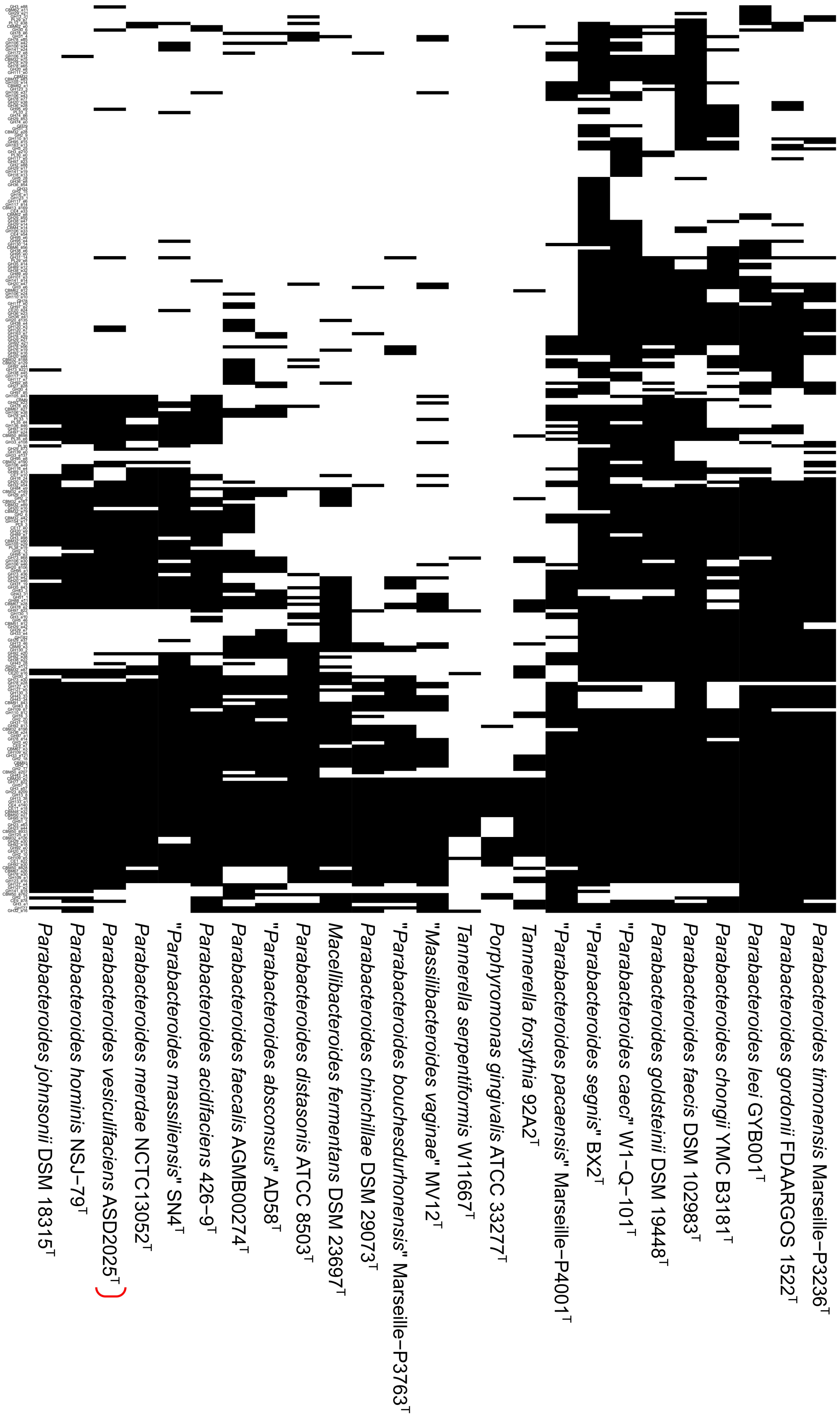

Figure S3 (part 2). Glycan degradation protein subfamilies in *Parabacteroides* and related genera. Each row corresponds to one of 555 subfamilies; black cells indicate the presence of a subfamily in a genome, while white cells indicate absence. Rows and columns were hierarchically clustered using Euclidean distance.

Table S1. Antibiotic susceptibility profile of the strain ASD2025^T, determined using a disk diffusion method on Fastidious anaerobe agar (Neogen, Lansing, MI, USA)) supplemented with 5% (v/v) defibrinated horse blood, incubated in an atmosphere of 85% N2/10% H2/5% CO2 at 37 °C for 17 h in anaerobic jars (Schuett-Biotec, Göttingen, Germany) with catalysts containing palladium-coated pellets.

Antibiotic	Amount of substance in disk, µg	Zone diameter breakpoints (mm) according to EUCAST recommendations*		ASD2025^T diameter of growth inhibition zone, mm**
		S≥	R<	
Imipenem	10	29	29	23.6 (23-25)
Meropenem	10	28	28	23.6 (22-26)
Ertapenem	10	23	23	19.7 (19-20)
Amoxycillin-clavulanate	20/10	14	14	11.3 (11-12)
Ampicillin-sulbactam	10/10	25	25	10 (7-12)
Piperacillin-tazobactam	30/6	24	24	20 (17-23)
Metronidazole	5	25	25	27.7 (27-28)
Clindamycin	2	10	10	20.6 (18-25)
Ceftazidime	30	-	-	5
Amikacin	10	-	-	5.7 (5-7)
Gentamicin	15	-	-	5
Vancomycin	5	-	-	6.7 (6-7)
Doxycycline hydrochloride	30	-	-	22.7 (22-24)
Azithromycin	30	-	-	5
Levofloxacin	5	-	-	22.7 (20-24)

*https://www.eucast.org/fileadmin/eucast/pdf/breakpoints/v_16.0_Breakpoint_Tables.pdf.

** Given as mean (range); calculated using three biological repeats. Measurements include the diameter of disk itself which is 5 mm, range is not given for the antibacterials with no growth inhibition zone outside of the disk.
